# Machine Learning and Data Mining Methods in Diabetes Research

**DOI:** 10.1016/j.csbj.2016.12.005

**Published:** 2017-01-08

**Authors:** Ioannis Kavakiotis, Olga Tsave, Athanasios Salifoglou, Nicos Maglaveras, Ioannis Vlahavas, Ioanna Chouvarda

**Affiliations:** a Department of Informatics, Aristotle University of Thessaloniki, Thessaloniki 54124, Greece; b Institute of Applied Biosciences, CERTH, Thessaloniki, Greece; c Laboratory of Inorganic Chemistry, Department of Chemical Engineering, Aristotle University of Thessaloniki, Thessaloniki 54124, Greece; d Lab of Computing and Medical Informatics, Medical School, Aristotle University of Thessaloniki, Thessaloniki 54124, Greece

**Keywords:** Machine learning, Data mining, Diabetes mellitus, Diabetic complications, Disease prediction models, Biomarker(s) identification

## Abstract

The remarkable advances in biotechnology and health sciences have led to a significant production of data, such as high throughput genetic data and clinical information, generated from large Electronic Health Records (EHRs). To this end, application of machine learning and data mining methods in biosciences is presently, more than ever before, vital and indispensable in efforts to transform intelligently all available information into valuable knowledge. Diabetes mellitus (DM) is defined as a group of metabolic disorders exerting significant pressure on human health worldwide. Extensive research in all aspects of diabetes (diagnosis, etiopathophysiology, therapy, etc.) has led to the generation of huge amounts of data. The aim of the present study is to conduct a systematic review of the applications of machine learning, data mining techniques and tools in the field of diabetes research with respect to a) Prediction and Diagnosis, b) Diabetic Complications, c) Genetic Background and Environment, and e) Health Care and Management with the first category appearing to be the most popular. A wide range of machine learning algorithms were employed. In general, 85% of those used were characterized by supervised learning approaches and 15% by unsupervised ones, and more specifically, association rules. Support vector machines (SVM) arise as the most successful and widely used algorithm. Concerning the type of data, clinical datasets were mainly used. The title applications in the selected articles project the usefulness of extracting valuable knowledge leading to new hypotheses targeting deeper understanding and further investigation in DM.

## 1 Introduction

Significant advances in biotechnology and more specifically high-throughput sequencing result incessantly in an easy and inexpensive data production, thereby ushering the science of applied biology into the area of big data [1], [2].

To date, besides high performance sequencing methods, there is a plethora of digital machines and sensors from various research fields generating data, including super-resolution digital microscopy, mass spectrometry, Magnetic Resonance Imagery (MRI), etc. Although these technologies produce a wealth of data, they do not provide any kind of analysis, interpretation or extraction of knowledge. To this end, the area of Biological Data Mining or otherwise Knowledge Discovery in Biological Data, is more than ever necessary and important. The primary objective is to delve into the rapidly accruing body of biological data and set the basis potentiating answers to fundamental questions in biology and medicine.

The power and effectiveness of these approaches are derived from the ability of commensurate methods to extract patterns and create models from data. The aforementioned fact is particularly significant in the big data era, especially when the dataset can reach terabytes or petabytes of data. Consequently, the abundance of data has strengthened considerably data-oriented research in biology. In such a hybrid field, one of the most important research applications is prognosis and diagnosis related to human-threatening and/or life quality reducing diseases. One such disease is diabetes mellitus (DM).

Applying machine learning and data mining methods in DM research is a key approach to utilizing large volumes of available diabetes-related data for extracting knowledge. The severe social impact of the specific disease renders DM one of the main priorities in medical science research, which inevitably generates huge amounts of data. Undoubtedly, therefore, machine learning and data mining approaches in DM are of great concern when it comes to diagnosis, management and other related clinical administration aspects. Hence, in the framework of this study, efforts were made to review the current literature on machine learning and data mining approaches in diabetes research.

The review is organized as follows: Section 2 provides the necessary background knowledge on machine learning (ML) and knowledge discovery in databases (KDD). Section 3 presents a concise presentation of the DM disease. Section 4 provides the methodological approach adopted, and Section 5, divided in five subsections, presents publications reviewed in the study. Section 6 presents a discussion, with Section 7 providing conclusions.

## 2 Machine Learning and Knowledge Discovery

Machine learning is the scientific field dealing with the ways in which machines learn from experience. For many scientists, the term “machine learning” is identical to the term “artificial intelligence”, given that the possibility of learning is the main characteristic of an entity called intelligent in the broadest sense of the word. The purpose of machine learning is the construction of computer systems that can adapt and learn from their experience [3]. A more detailed and formal definition of machine learning is given by Mitchel [4]: *A computer program is said to learn from experience E with respect to some class of tasks T and performance measure P, if its performance at tasks in T, as measured by P, improves with experience E*.

**Fig. 1:**
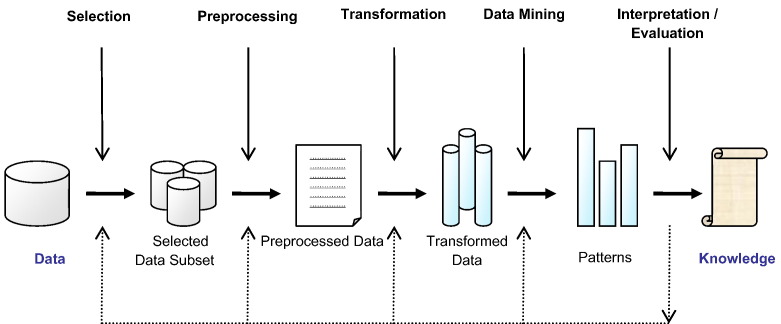
The basic steps of the KDD process.

Knowledge discovery in databases (KDD) is a field encompassing theories, methods and techniques, trying to make sense of data and extract useful knowledge from them. It is considered to be a multistep process (selection, preprocess, transformation, data mining, interpretation-evaluation) depicted in Fig. 1 [5]. The most important step in the entire KDD process is data mining, exemplifying the application of machine learning algorithms in analyzing data. A complete definition of KDD is given by Fayyad et al. [5]: *KDD is the nontrivial process identifying valid, novel, potentially useful, and ultimately understandable patterns in data*.

### 2.1 Categories of Machine Learning Tasks

Machine learning tasks are typically classified into three broad categories [6]. These are: a) supervised learning, in which the system infers a function from labeled training data, b) unsupervised learning, in which the learning system tries to infer the structure of unlabeled data, and c) reinforcement learning, in which the system interacts with a dynamic environment.

#### 2.1.1 Supervised Learning

In supervised learning, the system must “learn” inductively a function called target function, which is an expression of a model describing the data. The objective function is used to predict the value of a variable, called dependent variable or output variable, from a set of variables, called independent variables or input variables or characteristics or features. The set of possible input values of the function, i.e. its domain, are called instances. Each case is described by a set of characteristics (attributes or features). A subset of all cases, for which the output variable value is known, is called training data or examples. In order to infer the best target function, the learning system, given a training set, takes into consideration alternative functions, called hypothesis and denoted by *h*. In supervised learning, there are two kinds of learning tasks: classification and regression. Classification models try to predict distinct classes, such as e.g. blood groups, while regression models predict numerical values. Some of the most common techniques are Decision Trees (DT), Rule Learning, and Instance Based Learning (IBL), such as *k*-Nearest Neighbors (*k*-NN), Genetic Algorithms (GA), Artificial Neural Networks (ANN), and Support Vector Machines (SVM).

#### 2.1.2 Unsupervised Learning

In unsupervised learning, the system tries to discover the hidden structure of data or associations between variables. In that case, training data consists of instances without any corresponding labels.

##### 2.1.2.1 Association Rule Learning

*Association Rule Mining* appeared much later than machine learning and is subject to greater influence from the research area of databases. It was proposed in the early 1990s by Rakesh Agrawal [7] as a market basket analysis, in which the aim was to find correlations in the objects of a database. Based on the shopping cart example, association rules are of the form {X_1_, …, X_n_} → Y, which means that if you find all of X_1_, …, X_n_ in a cart it is *possible* to find Y. The most well-known association rule discovery algorithm is *Apriori*, proposed in 1994 by Rakesh Agrawal [8].

Although, association rule mining was first introduced as a market basket analysis tool, it has since become one of the most valuable tools for performing unsupervised exploratory data analysis over a wide range of research and commercial areas, including biology and bioinformatics. Some of the most well-known applications in biology and bioinformatics include biological sequence analysis, analysis of gene expression data and others. A thorough review of discovering frequent patterns and association rules from biological data, including algorithms and applications, can be found in [9].

##### 2.1.2.2 Clustering

Clusters are informative patterns occurring through clustering, i.e. the separation of a whole dataset into groups of data, so that instances belonging to the same group are as similar as possible and instances belonging to different groups differ as much as possible [10].

#### 2.1.3 Reinforcement Learning

The term *Reinforcement Learning* is a general term given to a family of techniques, in which the system attempts to learn through direct interaction with the environment so as to maximize some notion of cumulative reward [11]. It is important to mention that the system has no prior knowledge about the behavior of the environment and the only way to find out is through trial and failure (trial and error). Reinforcement learning is mainly applied to autonomous systems, due to its independence in relation to its environment.

### 2.2 Feature Selection

Feature selection is one of the most important processes of the KDD's data transformation step. It is defined as the process of selecting a subset of features from the feature space, which is more relevant to and informative for the construction of a model. The advantages of feature selection are many and relate to different aspects of data analysis, such as better visualization and understanding of data, reduction of computational time and duration of analysis, and better prediction accuracy [12], [13].

There are two main different approaches in the feature selection process. The first one is to make an independent assessment, based on general characteristics of data. Methods belonging to this approach are called filter methods, because the feature set is filtered out before model construction. The second approach is to use a machine learning algorithm to evaluate different subsets of features and finally select the one with the best performance on classification accuracy. The latter algorithm will be used in the end to build a predictive model. Methods in this category are called wrapper methods, because the arising algorithm wraps the whole feature selection process.

## 3 Diabetes Mellitus

Diabetes Mellitus (DM) is defined as a group of metabolic disorders mainly caused by abnormal insulin secretion and/or action [14]. Insulin deficiency results in elevated blood glucose levels (hyperglycemia) and impaired metabolism of carbohydrates, fat and proteins. DM is one of the most common endocrine disorders, affecting more than 200 million people worldwide. The onset of diabetes is estimated to rise dramatically in the upcoming years. DM can be divided into several distinct types. However, there are two major clinical types, type 1 diabetes (T1D) and type 2 diabetes (T2D), according to the etiopathology of the disorder. T2D appears to be the most common form of diabetes (90% of all diabetic patients), mainly characterized by insulin resistance. The main causes of T2D include lifestyle, physical activity, dietary habits and heredity, whereas T1D is thought to be due to autoimmunological destruction of the Langerhans islets hosting pancreatic-β cells. T1D affects almost 10% of all diabetic patients worldwide, with 10% of them ultimately developing idiopathic diabetes. Other forms of DM, classified on the basis of insulin secretion profile and/or onset, include Gestational Diabetes, endocrinopathies, MODY (Maturity Onset Diabetes of the Young), neonatal, mitochondrial, and pregnancy diabetes. The symptoms of DM include polyurea, polydipsia, and significant weight loss among others. Diagnosis depends on blood glucose levels (fasting plasma glucose = 7.0 mmol/L) [15].

DM progression is strongly linked to several complications, mainly due to chronic hyperglycemia. It is well-known that DM covers a wide range of heterogeneous pathophysiological conditions. The most common complications are divided into micro- and macro-vascular disorders, including diabetic nephropathy, retinopathy, neuropathy, diabetic coma and cardiovascular disease. Due to high DM mortality and morbidity as well as related disorders, prevention and treatment attracts broad and significant interest. Insulin administration is the main treatment for T1D, although insulin is also provided in certain cases of T2D patients, when hyperglycemia cannot be controlled through diet, weight loss, exercise and oral medication. Current medication targets primarily a) saving one's life and alleviating the disease symptoms, and b) prevention of long term diabetic complications and/or elimination of several risk factors, thereby increasing longevity. The most common anti-diabetic agents include sulfonylurea, metformin, alpha-glucosidase inhibitor, peptide analogs, non-sulfonylurea secretagogues, etc. [16]. The majority of the present anti-diabetic agents, however, exhibit numerous side-effects. In addition, insulin therapy is related to weight gain and hypoglycemic events. Hence, anti-diabetic drug design and discovery is of great concern and concurrently a research challenge [17], [18], [19], [20].

Although extensive research in DM has provided significant knowledge, over the past decades, on the a) etiopathology (genetic or environmental factors and cellular mechanisms), b) treatment, and c) screening and management of the disease, there is still much to be discovered, unraveled, clarified and delineated. Through such processes, diagnosis, prognostic evaluation of appropriate treatment and clinical administration could gain significant ground toward medical handling of the disease. In such an effort, reliance on a large and fast increasing body of research and clinical data serves to establish a significant basis for safe diagnosis and follow-up treatment. Thus, data mining and machine learning emerge as key processes, contributing decisively to the decision-making clinician. The aspiration, therefore, is to link data assessment to diagnosis and appropriate decision-making in drug administration.

## 4 Methods

Extensive efforts were made to identify articles employing machine learning and data mining techniques on diabetes research. Two databases were searched (15 July 2016): the one extensively used in biomedical sciences, PubMed and the DBLP Computer Science Bibliography, containing more than 3.4 million journal articles, conference papers, and other publications on computer science (July 2016) [21]. The main reason behind the utilization of DBLP was that there are certain high impact international scientific journals in the computer science field that are not indexed by PubMed, although in some cases, the proposed published methods are applied on biomedical datasets.

As mentioned previously, there is a close relationship between the terms machine learning and data mining, with the latter being more generic. Thus, often, in scientific literature, machine learning methods are called data mining methods. To overcome that and be more accurate in finding all related articles, two searches were performed in PubMed, based on the following queries: a) “Machine Learning” AND “Diabetes” (QUERY_1), and b) “Data Mining” AND “Diabetes” (QUERY_2). Although PubMed launches searches on the title, abstracts and keywords of an article, DBLP conducts searches only on the title. In view of this fact, searches in DBLP were limited only to “Diabetes” query (QUERY_3), since machine learning and data mining are too broad terms to be found on a computer science article title.

Due to the vast amount of articles returned from the three queries (QUERY_1: 139, QUERY_2: 268, and QUERY_3: 880), our search was limited to articles published over the past five years (automatically in PubMed and manually in DBLP), thereby narrowing down significantly the retrieved collection (QUERY_1: 110, QUERY_2: 184, and QUERY_3: 248). It is important to mention that the huge collection of articles, retrieved through DBLP, was due to the fact that articles were not only limited to the machine learning and data mining fields but also covered the broader computer science field in general.

**Fig. 2:**
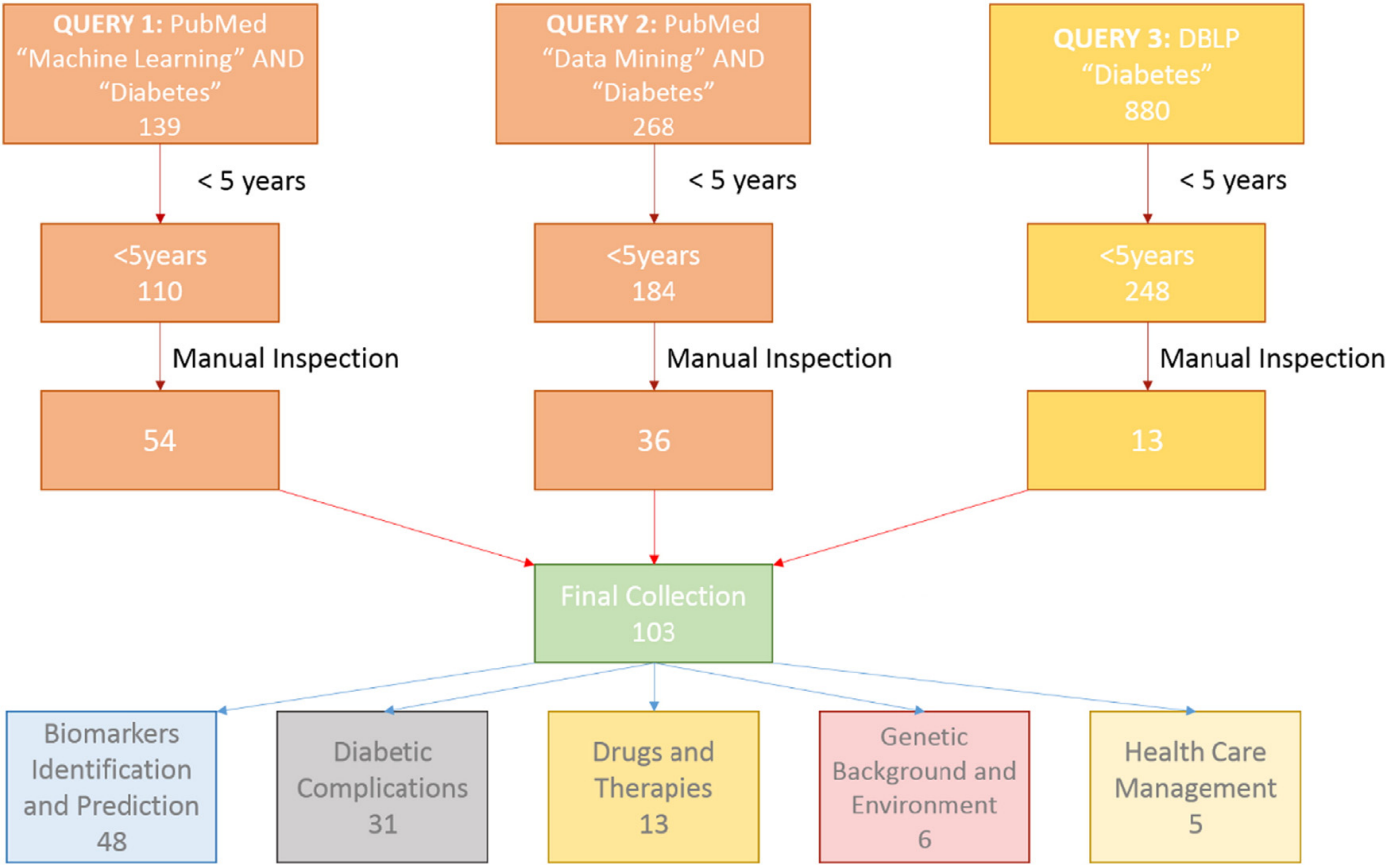
Literature selection and classification process.

**Fig. 3:**
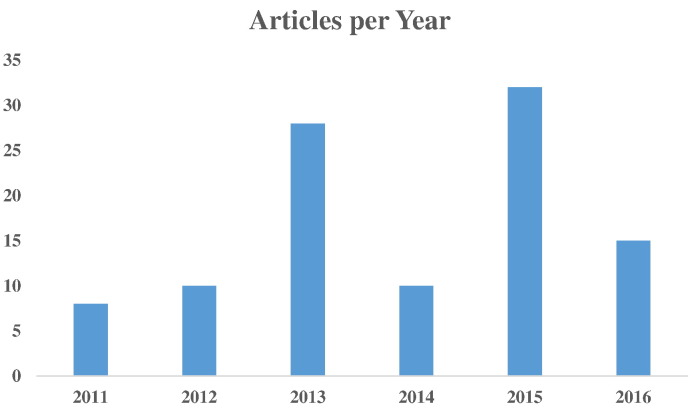
Articles per year in the collection employed.

The next step was manual inspection of all recovered articles. The common purpose of this manual inspection for all three queries was to initially assess their relationship to Diabetes research. Moreover, for QUERY_2, manual inspection was performed to exclude articles that didn't contain machine learning methods; for instance, articles with simple statistical analyses. Lastly, concerning QUERY_3, the purpose of manual inspection was twofold. Firstly, to find all articles related to machine learning and secondly to identify and merge overlaps among queries, i.e. articles already indexed by PubMed, which included the vast majority of them. Manual inspection narrowed down even more the collection (QUERY_1: 54, QUERY_2: 36, and QUERY_3: 13), thereby resulting in a final collection of 103 articles. These articles were classified into the following five categories: Biomarker Prediction and Diagnosis in DM; Diabetic Complications; Drugs and Therapies; Genetic Background and Environment, and Health Care Management. The entire article selection process is illustrated in a workflow (Fig. 2), with the number of publications per year being depicted in Fig. 3.

Since the area of data mining and machine learning applied to Diabetes is very wide, it is hard to include every single research study. The selected methodology was employed in an effort to present only the latest research efforts in DM. In this term, the current collection consists of research work conducted the last five years. Moreover, in the present study, specific keywords were used such as “machine learning” and “data mining”. However, there are several additional keywords that could possibly be used concerning specific algorithms, e.g. neural networks or specific tasks (i.e. predictive modeling), that belong to the field of machine learning and data mining without mentioning the current terms. In that sense, the results of this research are not exhaustive.

## 5 DM Through Machine Learning and Data Mining

This section presents key papers of the study.

### 5.1 Biomarker Identification and Prediction of DM

A large number of factors are known to be important in the development and progression of DM. Obesity stands as a major risk factor, especially in T2D, given the strong causal relationship between that and the onset of DM [22]. DM diagnosis is carried out through several tests [α-glycate hemoglobin (A1C) test, random blood sugar test, fasting sugar test or oral glucose tolerance test]. There is evidence that in both T1D and T2D, early diagnosis and prediction of the onset of the disease are vital to the a) retardation of the progression of the disease, b) targeted selection of the medication, c) prolonging life expectancy, symptom alleviation, and d) onset of related complications.

Biomarkers (e.g. biological molecules) are measurable indicators of a certain condition representing health and disease states. Typically, biomarkers are a) measured in body fluids (blood, saliva or urine), b) encountered and thus determined independent of their etiopathogenic mechanistic pathway, and c) used to monitor clinical and subclinical disease burden and response to treatments. Biomarkers can be direct ending points of the disease itself or indirect indexes of other complications. Current technologies, such as metabolomics, proteomics, and genomics contribute to the development of a plethora of new biomarkers. In the case of DM, biomarkers may reflect the presence and severity of hyperglycemia or presence and severity of the related complications in diabetes [23].

The current section is divided into two main categories, which include cases where a) diagnostic and predictive markers are employed or new biomarkers are introduced, and b) disease prediction takes place, although this task is always performed to evaluate the predictive accuracy of the identified biomarkers.

#### 5.1.1 Diagnostic and Predictive Markers

The first category deals with biomarker discovery, which is a task mainly performed through feature selection techniques [24], [25], [26], [27], [28], [29], [30], [31], [32], [33], [34]. Following a feature selection step, a classification algorithm is employed to assess the prediction accuracy of the selected features.

Firstly, established methods have been used in the biomarker evaluation issue. In [25], the authors used a clinical dataset comprised of 803 prediabetic females with 55 features and compared several common feature selection algorithms (both wrapper and filter methods) to predict DM. They concluded that the best overall performance had been achieved through wrapper methods. Moreover, among the filter methods used, symmetrical uncertainty achieved the best prediction accuracy. In another work, using established methods, Georga et al. [28] applied Random Forest (RF) [35] and RReliefF [36] to evaluate a number of features, with respect to their ability to predict the short-term subcutaneous glucose concentrations. In [31], authors combined gas chromatography–mass spectrometry (GC/MS) profiling with Random Forest, in an effort to explore relationships between 5′-AMP-activated protein kinase AMPK and DM. Jelinek et al. [24] investigated whether additional biomarkers could be used together with HbA1c to improve diagnostic accuracy in T2D, in case HbA1c levels are below or equal to the current cut-off of 6.5%. They concluded that both 8-hydroxy-2-deoxyguanosine (8-OhdG), an oxidative stress marker, and interleukin-6 (IL-6) improved classification accuracy.

Novel methods have also been proposed to deal with features in diabetic patient data. Improved electromagnetism-like mechanism (IEM) algorithm [26] was proposed for feature selection. It combines electromagnetism-like mechanism (EM) algorithm with the nearest neighbor classifier [37] and opposite sign test (OST) [38] as the local search. A completely different approach, dealing with features in a diabetic clinical dataset, is proposed in [33]. Authors used genetic programming to generate new features from existing ones, without prior knowledge of the probability distribution. Sideris et al. proposed a novel, clustering-based (hierarchical clustering) feature extraction framework, using disease diagnostic information [34]. Their methodology produced clusters to be used as features for patient severity of condition and patient readmission risk prediction.

Finally, work on high-dimensional data was presented in [27]. Feature selection is a very challenging task, when performed in high-dimensional data such as genomic data. Cai et al. applied a feature selection method, called iterative sure independence screening (ISIS) [39] for gene profiles obtained from metagenome sequencing in Chinese/European cohorts, achieving 0.97/0.99 accuracy following selection of 48/24 meta-markers.

#### 5.1.2 Prediction of DM

The second category deals with disease prediction and diagnosis [40], [41], [42], [43], [44], [45], [46], [47], [48], [49], [50], [51], [52], [53], [54], [55], [56], [57], [58], [59], [60], [61], [62], [63], [64], [65], [66], [67], [68], [69], [70], [71], [72], [73], [74], [75], [76]. Numerous algorithms and different approaches have been applied, such as traditional machine learning algorithms, ensemble learning approaches and association rule learning in order to achieve the best classification accuracy. Most noted among the aforementioned ones are the following:

Calisir and Dogantekin proposed LDA–MWSVM, a system for diabetes diagnosis [66]. The system performs feature extraction and reduction using the Linear Discriminant Analysis (LDA) method, followed by classification using the Morlet Wavelet Support Vector Machine (MWSVM) classifier. Gangji and Abadeh [65] proposed an Ant Colony-based classification system to extract a set of fuzzy rules, named FCS-ANTMINER, for diabetes diagnosis. In [68], authors dealt with glucose prediction as a multivariate regression problem utilizing Support Vector Regression (SVR). Agarwal et al. [48] utilized semi-automatically labeled training sets to create phenotype models via machine learning methods. In [76], authors proposed a fuzzy ontology-based Case-based reasoning (CBR) framework, mimicking expert thinking, further tested on diabetes diagnosis problems. In [58], authors performed an evaluation of Stream Mining Classifiers for Real-time Clinical Decision Support Systems.

With respect to high dimensional datasets, Razavian et al. [44] used a dataset containing 4.1 million individuals and 42K variables from administrative claims, pharmacy records, healthcare utilization, and laboratory results between 2005 and 2009, to build predictive models (based on logistic regression) for different onsets of T2D prediction.

A completely different study is presented in [40]. Authors built disease progression models, taking into account trajectories, i.e. the sequence of events leading to a state. When applied to Diabetes data, they identified a typical trajectory from hyperlipidemia (HLD) to hypertension (HTN), impaired fasting glucose (IFG), and T2D.

Ensemble approaches, which use multiple learning algorithms, have proven to be an effective way of improving classification accuracy. The specific approaches have also been used in DM prediction [50], [52], [53], [69]. Anderson et al. used a Bayesian scoring algorithm to explore the model space [50]. In [52], authors proposed an ensemble framework with multi-layer classification, using enhanced bagging and optimized weighting, combining seven heterogeneous classifiers. In [53], authors used Rotation Forest (RF), a newly proposed ensemble algorithm, to combine 30 machine learning algorithms. Finally, Han et al. presented an ensemble learning approach, which turns the “black box” of SVM decisions into comprehensible and transparent rules [69].

Association Rules are mainly employed to identify associations between risk factors in an interpretable form [71], [72], [73], [74]. In [71], authors applied association rules to detect combinations of variables or predictors frequently occurring together in diabetic patients. Simon et al. proposed Survival Association Rule (SAR) Mining [72], an extension to traditional Association Rule Mining, which can handle survival outcomes, make adjustment for confounders and incorporate dosage effects. In [73], authors reviewed four association rule set summarization techniques and proposed extensions, in order to deal with the large number of rules, mined from ARM applied to high dimensional EMR data. Finally, Batal et al. utilized temporal pattern mining for discovering predictive patterns in complex multivariate time series data, to improve performance of current classifiers [74].

### 5.2 Diabetic Complications

As mentioned above, the main pathophysiological feature in DM is hyperglycemia. In addition to normal glucose metabolism, prevention of complications due to elevated glucose levels is of great concern. Generally, the harmful effects of hyperglycemia are divided into a) macrovascular complications, such as coronary artery disease, peripheral arterial disease, and stroke and, b) microvascular complications that include diabetic neuropathy, nephropathy, and retinopathy [77]. The direct and indirect effects of hyperglycemia are the main source of morbidity and mortality in both T1D and T2D. Large prospective clinical studies show a strong relationship between glycemia and diabetic microvascular complications in both T1D and T2D. Diabetic complications can also be classified according to their severity and time of onset. In these terms, acute diabetic complications include: diabetic ketoacidosis, hypoglycemia, diabetic coma, erectile dysfunction, respiratory infections and periodontal disease. Chronic diabetic complications include: heart failure, diabetic neuropathy, nephropathy, retinopathy, and diabetic foot. Moreover, both insulin resistance and hyperglycemia have been implicated in the pathogenesis of diabetic dyslipidemia. It is worth noting that DM complications are far less common and severe in people with well-controlled blood glucose levels. Many of those complications have been studied through machine learning and data mining applications [78], [79], [80], [81], [82], [83], [84], [85], [87], [88], [89], [90], [92], [94], [95], [96], [97].

In a more general aspect, Lagani et al. targeted several diabetic complications, such as cardiovascular diseases (CVD), hypoglycemia, ketoacidosis, microalbuminuria, proteinuria, neuropathy, and retinopathy [78], [79]. In [78], in an effort to identify the smallest set of clinical parameters with the best predictive accuracy, involving the aforementioned diabetic complications, a set of predictive models was used that had been developed through data mining and machine learning approaches. In [80], authors used two distinct data sources (drug purchase and administrative information) to exploit temporal data mining techniques and improve risk stratification of diabetic complications.

In the case of nephropathy, Huang et al. employed a Decision Tree-based prediction tool that combines both genetic and clinical features in order to identify diabetic nephropathy in patients with T2D [81]. Leung et al. compared several machine learning methods that include partial least square regression, classification and regression tree, the C5.0 Decision Tree, Random Forest, naïve Bayes, neural networks and support vector machines [82]. The dataset used consists of both genetic (Single Nucleotide Polymorphisms — SNPs) and clinical data. Age, age of diagnosis, systolic blood pressure and genetic polymorphisms of uteroglobin and lipid metabolism arose as the most efficient predictors.

Similarly, in the case of neuropathy, DuBrava et al. used Random Forest (RF) in order to select specific features targeting prediction of diabetic peripheral neuropathy (DPN) [83]. Based on relevance, the features chosen were Charlson Comorbidity Index score (100%), age (37.1%), number of pre-index procedures and services (29.7%), number of pre-index outpatient prescriptions (24.2%), number of pre-index outpatient visits (18.3%), number of pre-index laboratory visits (16.9%), number of pre-index outpatient office visits (12.1%), number of inpatient prescriptions (5.9%), and number of pain-related medication prescriptions (4.4%). The overall accuracy of the model reached 89%. The Database of the diabetes screening research initiative (DiScRi) [113] was used in [84], [85] to predict Cardiovascular Autonomic Neuropathy (CAN). Staniery et al., used decision tree and optimal decision path finder (ODPF) to find the optimal sequences of Ewing tests to predict CAN, whereas Abawajy et al. used regression and meta-regression, in combination with the Ewing formula, to identify the classes in CAN, thus overcoming the problem of missing data.

Although Alzheimer's disease is a chronic neurodegenerative disease, seemingly not related to DM, several studies support the fact DM and AD have a strong causal relationship [86]. Alzheimer's disease is often referred to as “type 3” diabetes. In [87], authors delved into the relationship between DM and AD via semantic data mining. Following extensive analysis of several paper abstracts, they managed to identify genes related to both diseases. Efforts were also made to construct an interaction network in order to identify existing links (genes and molecules) in the network.

In [88], authors developed a predictive model exploiting data from two safety-net clinical trials that target comorbid depression, which could be considered as a diabetic complication, among patients with DM. In addition, in [89], authors tried to investigate the effectiveness of e-nose technology, using common classifiers, to predict single- and poly-microbial species targeted for diabetic foot infection. Rau et al. [90], also, developed a model to predict liver cancer within six years following T2D diagnosis. A dataset comprised of 2060 cases, was divided into two groups, encompassing patients a) diagnosed with liver cancer after diabetes, and b) with diabetes, but no liver cancer.

Heart-related abnormalities are considered as common diabetic complications [91]. It is worth noting that there's a significant link between diabetes, heart disease, and stroke. In fact, two out of three people with diabetes die from heart disease or stroke, also called cardiovascular disease. In [92], researchers developed a hybrid approach, partially based on conditional random field classifier, to extract related information on heart disease risk factors from longitudinal unstructured EHRs.

Hypoglycemia, reflecting low blood sugar levels, arises mainly due to anti-diabetic treatment and has a great impact among DM patients [93]. Machine learning methods, such as Random Forest, support vector machines (SVM), k-nearest neighbor, and naïve Bayes, were used by Sudharsan B et al. [94] to predict hypoglycemia among patients with T2D, whereas support vector regression was used by Georga et al. [95] for the same reason. Moreover, a comparison of already published algorithms was reported by Jensen [96] in the same framework.

Intentional insulin treatment omission is an inappropriate compensatory behavior, occurring mainly in female patients with T1D, who omit or restrict their required insulin doses in order to lose weight. Although that does not occur as a diabetic complication but rather as a compensatory behavior, diagnosis of this underlying disorder is of great concern. In [97], authors used decision trees to analyze clinical and laboratory data for the prediction of intentional insulin omission for intentional weight loss.

Diabetic Retinopathy (DR) is an eye disease, occurring in people with either T1D or T2D. The longer a patient has diabetes the higher the risk of developing the specific pathophysiological condition. DR usually exhibits early warning signs and is characterized as a major diabetic complication [98]. DR can be divided into two main stages: a) NPDR (non-proliferative DR), and b) PDR (proliferative DR). Given the considerable impact of the current complication on patient lifestyle as well as society, numerous efforts have targeted accurate prediction of the disease onset in an effort to prevent progression.

Considering data mining and machine learning approaches, DR is the most studied field, mainly based on image processing techniques [100], [101], [102], [103], [104], [105], [106], [107], [108], [109], [110], [111], [112]. A comprehensive review on computational methods for diabetic retinopathy was published in 2013 [99]. Interestingly, in [100], [101], data acquisition was also based on proteomic analyses. Specifically, Torok et al. developed a method, in which different types of data (results from tear fluid proteomics analysis and digital micro aneurysm detection on fundus images) were used as input in a Gradient Boosting Machine for DR screening, whereas Jin et al. performed comprehensive proteomics analysis to identify biomarkers for DR, concluding that a four protein biomarker panel (APO4, C7, CLU, and ITIH2) is capable of detecting early stages of the disease. Oh et al. [102] reported the first attempt in predicting DR using least absolute shrinkage and selection operator (LASSO) exploiting health record data. Moreover, Ibrahim et al. [103] used a data adaptive neuro fuzzy inference classifier to predict diabetes maculopathy. Roychowdhury et al. [104] targeted the degree of severity in DR, using a computer-aided screening system (DREAM) that analyzes fundus images with varying illumination and fields of view via machine learning approaches. A two phase method, Diabetic Fundus Image Recuperation (DFIR), was used in [105] for DR prediction. The first phase performs feature selection on digital retinal fundus images. The second phase employs a support vector machine for the prediction. A different aspect to the DR problem was investigated by Pires et al. [106]. In that case, a method for assessing the need for referral was developed, based on the identification of DR-related lesions in retinal images. Finally, Giancardo et al. proposed a methodology for Diabetic macular edema prediction, which is a common vision-threatening complication of DR [107]. Zhang et al. proposed a method for detecting DM and Non proliferative DR (early event) using tongue color, texture, and geometry features [110].

### 5.3 Drugs and Therapies

People with both types of diabetes need medication to help maintain normal blood sugar levels. The type of medication clearly depends on the type of diabetes. Insulin is the most common type of medication employed in T1D treatment and also used to treat T2D in some cases, depending on the severity of insulin depletion. At present, the majority of current therapies for T2D rely mainly on a number of approaches intending to reduce hyperglycemia. Such factors include sulfonylureas, metformins, PPAR-γ agonists (peroxisome proliferator-activated receptor-γ), α-glucosidase inhibitors, and others. Although diabetes constitutes a worldwide epidemic, with significant efforts targeting effective drug design and therapeutic protocols, most current therapies for this disease were developed in the absence of defined molecular targets or full delineation of the disease pathogenesis. Given the a) numerous side-effects of the present therapeutic protocols, and b) rapidly accruing knowledge on pathophysiological mechanisms, drug design and discovery stand as a great challenge in current research οn diabetes. Intensive study of the mechanisms of action of older drugs has provided further validation of several recently identified drug targets. Further efforts in this direction are likely to be fruitful. In the era of post-genomic drug development, extracting and applying knowledge from biochemical, chemical, biological, and clinical data is one of the most interesting challenges facing the pharmaceutical industry. By the same token, data mining techniques can help a) recommend and improve effective medication, b) predict and suggest more personalized medications, c) design more effective blood glucose lowering factors, d) improve insulin planning and dosage, and e) implement drug administration in a more specific manner.

Sequential pattern mining techniques are used to mine patterns from data, where values are delivered in a sequence. Thus, such techniques are suitable in predicting the sequence of medications to be prescribed for a patient. Wright et al. used sequential pattern mining (CSPADE algorithm) to identify temporal relationships among medication prescriptions and finally predict the follow-up medication to be prescribed for a patient [114]. Also, Deja et al. used differential sequence patterns to imprint deviations observed in patient blood glucose levels and the amount of insulin dose to improve physician therapy recommendations [115].

In addition, to improve dosage planning, case-based reasoning was used to optimize the appropriate and effective dose of insulin in T1D [116]. By the same token, Karahoca and Tunga [117] used High Dimensional Model Representation (HDMR) to manage the drug dosage planning process in T2D. Moreover, taking into consideration patient behavior in relation to patient care, Namayanja and Janeja used clustering techniques to improve insulin treatment in T2D patients [118].

To search for novel anti-diabetic agents, the potency of inhibiting DPP4 was employed in [119], through decision tree classifier based on thirteen physicochemical properties, including molecular weight, total energy of a molecule, and topological polar surface area. A QSAR model was also used to assess flavonoid inhibitory effects on AR activity as a potent treatment for diabetes, using artificial neural networks [120]. In [121], a novel method was proposed, based on association rule mining, to discover relationships between statin (reductase inhibitors, medication for cardiovascular disease) use and diabetes. In addition, in [122], the study aimed at determining whether data mining methodologies could identify reproducible predictors of dapagliflozin-specific treatment response in the phase 3 clinical program dataset.

Liu et al. performed feature selection using wrapper and filter approaches on a 258 feature set in order to improve classification accuracy for medication recommendation in T2D using K-Nearest Neighbor [123].

Gastrointestinal surgery is considered as an alternative beneficial treatment for morbidly obese T2D patients. Authors in [124], [125] targeted selection of markers for the prediction of successful T2D remission, following gastrointestinal surgery via artificial neural networks.

Postprandial hyperglycemia is considered as a global threat for both prediabetes and T2D. However, the current dietary methods for managing blood glucose levels exhibit limited efficacy. Zeevi et al. developed a machine learning algorithm that takes into account blood parameters, dietary habits, anthropometrics, physical activity, and gut microbiota to predict personalized postprandial glycemic response to real-life meals [126].

### 5.4 Genetic Background and Environment

Both type 1 and 2 diabetes as well as other rare forms of diabetes that are directly inherited, including MODY and diabetes due to mutations in mitochondrial DNA, are caused by a combination of genetic and environmental risk factors. Unlike some traits, diabetes does not seem to be inherited in a simple pattern. Undoubtedly, however, some people are born prone to developing diabetes more so than others. Several epidemiological patterns suggest that environmental factors contribute to the etiology of T1D. Interestingly, the recent elevated number of T1D incidents projects a changing global environment, which acts either as initiator and/or accelerator of beta cell autoimmunity rather than variation in the gene pool. Several genetic factors are involved in the development of the disease [127]. There is evidence that more than twenty regions of the genome are involved in the genetic susceptibility to T1D. The genes most strongly associated with T1D are located in the HLA region of chromosome 6 [128]. Similar to T1D, T2D has a strong genetic component. To date, more than 50 candidate genes for T2D have been investigated in various populations worldwide. Candidate genes are selected due to their interference with pancreatic beta cell function, insulin mode of action, glucose metabolism and/or other risk factors. It is a fact that advances in genotyping technology, over the past few years, have facilitated rapid progress in large-scale genetic studies. Identification of a large number of novel genetic variants increasing susceptibility diabetes and related traits opened up opportunities, not existing thus far, to associate this genetic information with clinical practice and possibly improve risk prediction. However, available data to date do not yet provide convincing evidence supporting use of genetic screening in the prediction of diabetes.

The human leukocyte antigen (HLA) system is a gene complex encoding the major histocompatibility complex (MHC) proteins in humans. HLA types are inherited and some of them are linked to autoimmune disorders and/or other diseases, including T1D diabetes. This fact has also been emphasized by recent genome-wide association studies. Zhao et al. attempted to reduce genetic association to practice through an HLA-based disease predictive model [129]. The authors managed to overcome the burden of low-predicting accuracy by using highly polymorphic genes as predictors. They proposed a methodology, which treats complex HLA genotypes as “objects”, and built predictive models for T1D using eight HLA genes (HLA-DRB1, HLA-DRB3, HLA-DRB4, HLA-DRB5, HLA-DQA1, HLA-DQB1, HLA-DPA1, and HLA-DPB1). By the same token, authors in [130] analyzed 19,035 SNPs of 10,579 subjects and selected as few as three SNPs to predict HLA-DR/DQ types relevant to T1D.

Even pleiotropic genes have a strong impact on DM onset and progression. It's a fact that pleiotropic genes cannot be easily associated with important diseases. To this end, Park et al. developed an association rule mining-based method to discover patterns of multiple phenotypic associations over 52 anthropometric and biochemical traits [131]. The discovered patterns were then used to identify genetic markers that can be associated with multivariate phenotypes.

It is worth mentioning that in [132], a meta-analysis study was conducted, where a collection of gene expression datasets of pancreatic beta-cells, conditioned in an environment resembling T1D induced apoptosis, such as exposure to pro-inflammatory cytokines, in order to identify relevant and differentially expressed genes. The specific genes were then characterized according to their function and prior literature-based information to build temporal regulatory networks. Moreover, biological experiments were carried out revealing that inhibition of two of the most relevant genes (RIPK2 and ELF3), previously unknown in T1D literature, have a certain impact on apoptosis.

On the other hand, Lee et al. used various classification algorithms, such as SVM and logistic regression, to predict T2D by employing 499 known SNPs from 87 T2D-related genes [133]. Finally, in [134], authors used support vector machines to predict tyrosine kinase ligand-receptor pairs from their amino acid sequences. More specifically, authors initially collected tyrosine kinase ligand-receptor pairs from the Database of Interacting Proteins (DIP) and UniProtKB, and after a filtering process, used them as a dataset for the assessment of predictive performance. Identification of the interacting partner of tyrosine kinase ligand-receptor, provides a deeper delineation of cellular-combined processes.

### 5.5 Health Care Management Systems

As mentioned above, the prevalence of diabetes for all age groups worldwide was estimated to be 2.8% in 2000 and 4.4% in 2030 [135]. The total number of people diagnosed with diabetes is projected to rise from 171 million in 2000 to 366 million by 2030. It is a grim fact that the majority of healthcare institutions in many countries spend billions of dollars on Diabetes health care. Given the impact of the disease, efforts are presently made to assess existing data in order to manage public health issues, such as hospitalization cost or medication.

In [136], authors presented a method to predict health care spending in ambulatory diabetes patients. In their method, authors used patterns extracted from health-related quality of life (HRQOL) inventories and electronic medical records and developed a hybrid approach based on Natural Language Processing and machine learning for the prediction models.

Nimmagadda et al. [137] developed a robust back-end application for web-based patient–doctor consultations and e-Health care, based on ontology-based multidimensional data warehousing and mining methodologies, while Renard et al. [138] developed DIABECOLUX, an algorithm for the prediction of treated T2D patients via health insurance claims, when no diagnosis code is available. Similarly, in [139], data from electronic health records and financial billing systems were used to produce integrated patient-based datasets. Mining of such data through probabilistic clustering methodologies allows assessment of the health and financial risk status, subsequently aiding in taking the appropriate proactive actions. Ultimately, Lee and Giaraud-Carrier [140] aimed at mining a huge collection of data, through association rules and clustering techniques, to support evidence-based medicine. Data were obtained from The National Health and Nutrition Examination Survey (NHANES), which is a program trying to assess health and nutritional status of adults and children in the United States.

## 6 Discussion

In the present study, the recent literature was reviewed with respect to applications of machine learning and data mining methods in Diabetes research. The first sections describe briefly the two main research fields involved (machine learning, knowledge discovery in databases and Diabetes), pointing out the necessity of intelligent applications in improving the quality and effectiveness of decision making in DM.

**Fig. 4:**
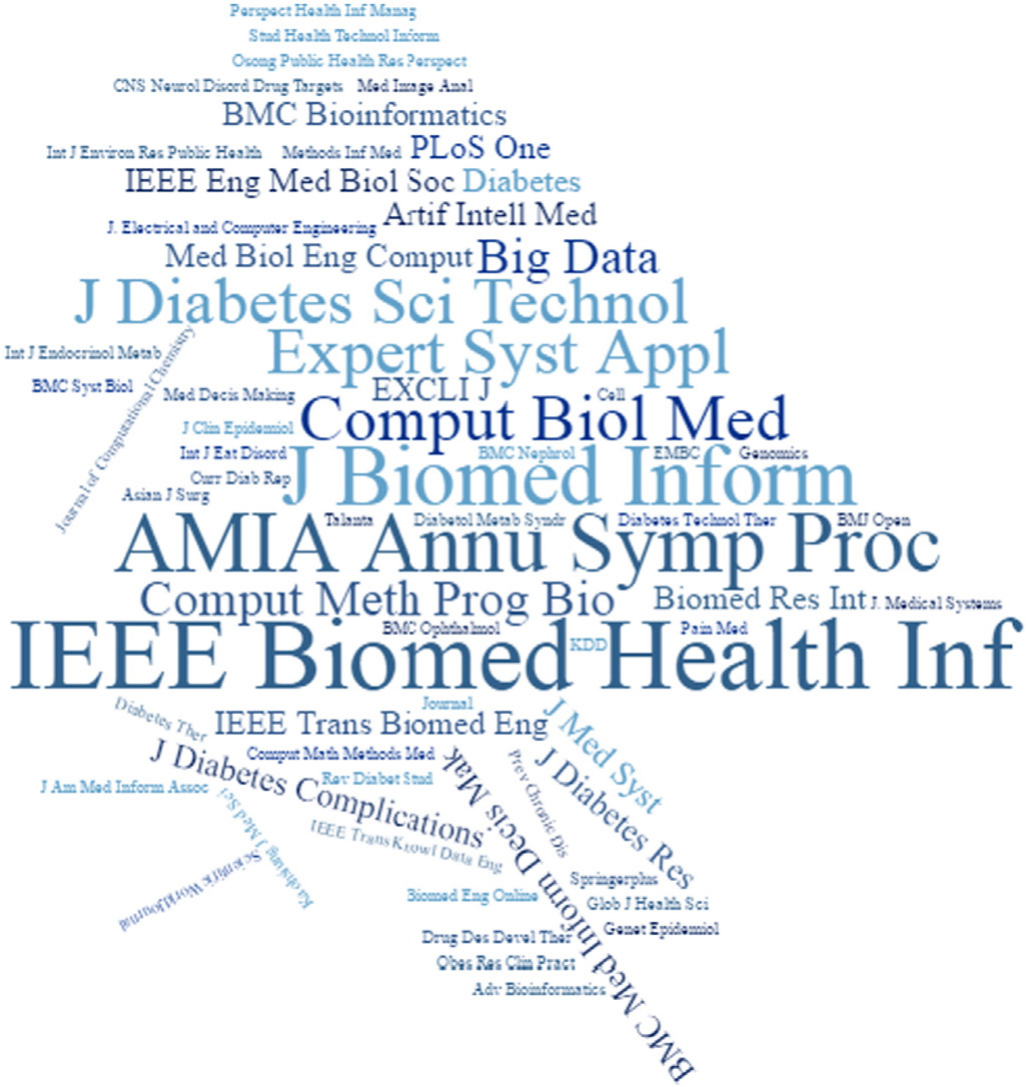
Distribution of articles in scientific journals.

Following creation of the assembled article collection (for methodology details vide supra), each article was categorized accordingly in one of the title groups (descending number of papers), thus covering to a great extent significant diabetes research fields, i.e. Biomarker Prediction and Diagnosis in DM, Diabetic Complications, Drugs and Therapies, Genetic Background and Environment, and Health Care Management. The current articles were published in several scientific journals that deal with distinct and wide fields of interest, including bioinformatics, biomedical engineering and diabetes. In Fig. 4, the scientific journals are presented in line with their appearance in the present collection, whereas Fig. 3 depicts the number of articles published per year.

The specific article categorization was carried out based on the content of the retrieved articles. The most popular category was the Biomarker Prediction and Diagnosis of DM, thematically revolving around efforts to discover and suggest novel biomarkers and finally predict key aspects of the disease, such as its onset. Since the undertaken research reflects a data-driven process, the arising gaps and limitations in machine learning research in DM are closely related to the availability of data. Clinical, diagnostic data and EHR are plentiful due to low cost of their retrieval, in contrast to other types of data, such as biological, which are more difficult and expensive to generate and therefore less available to the scientific community. That partially justifies the extensive research effort on specific topics, such as retinopathy. Moreover, there is complete lack of data concerning a) lifestyle and behavior, b) inheritance, and c) linkage with other pathophysiological conditions e.g. Alzheimer's disease.

### 6.1 Computational Insight Into Diabetes Research

When it comes to machine learning and data mining, significant conclusions are drawn through the present detailed account. It is worth mentioning that the vast majority of the reported articles enhanced classification accuracy, above 80%, in the prediction of DM. With regard to the prediction task itself, almost all of the common known classification algorithms have been employed. However, the most commonly used ones are SVM, ANN, and DT. It should be mentioned that SVM rises as the most successful algorithm in both biological and clinical datasets in DM. A great deal of articles (~ 85%) used the supervised learning approaches, i.e. in classification and regression tasks. In the remaining 15%, association rules were employed mainly to study associations between biomarkers. More specifically, concerning the part dealing with the evaluation task, in all reported research reports, the identified subsets of biomarkers (features) were evaluated through appropriate procedures, such as splitting the dataset into train and test set or via cross-validation. By analogy, the same approaches have been followed in DM prediction.

Worth emphasizing is the fact that in many studies, after the feature/biomarker selection, researchers have performed comparative analysis on different machine learning algorithms in order to assess their predictive performance and finally choose the most efficient one(s). To this end, this should be the baseline of practice in every study to be carried out, taking into account that several characteristics of the dataset, such as dimensionality, low number of instances compared to number of features or even the type of the dataset itself (genetic or clinical), can affect significantly the performance of the algorithm. Hence, an algorithm with the best performance in one dataset could easily have lower prediction accuracy compared to other algorithms in different datasets. Table 1 represents studies that compare more than five machine learning algorithms in various biological and clinical datasets. SVM exhibited the best performance with regard to classification accuracy or the Area Under the Curve (AUC). Moreover, many times in KDD, algorithms that produce interpretable results, are preferably used, although they are not necessarily state-of-the-art. The aforementioned fact explains, at least partially, the wide use of decision trees in the literature. The overall results project that a wide variety of algorithms and techniques are used in DM research. Obviously, different machine learning tasks are used in different scientific questions, such as prediction on DM or association among biomarkers. To this end, classification and regression techniques are used for prediction tasks, such as prediction of glucose levels and association rules in the case of dependencies between biomarkers. Interestingly, for each machine learning task, a variety of algorithms have been used in the literature. The reason behind that is likely the fact that the accuracy of an algorithm depends heavily on the type of data (dimensionality, origin and kind). Accordingly, a great effort in research relies on the preprocessing of data, such as feature selection and then various algorithms are applied to the processed data in order to identify the most successful one for the particular dataset.

Furthermore, it is imperative for machine learning studies that a dataset be sufficiently large for the algorithm to be trained appropriately. Although biomedical sciences have entered the era of big data for several reasons, such as low cost of next generation sequencing or unified EHRs, datasets with great variability in size are very common in DM research. In that respect, what should be stressed out is the danger of a) producing low quality results, and b) concomitantly having the entire KDD process finally extract low quality of knowledge, when a small amount of data is employed.

### 6.2 Computational Interfacing With Diabetes Mellitus

Potential gains of early detection of a disease, in this case DM, in addition to the assessment of possible risk factors, include a) significant prolongation and quality of life, pertaining to the reduction of severity and frequency of a disease state and/or prevention and delay of its complications, and b) reduction of health care cost, as a consequence of reduced care linked to hospitalization of patients. In this context, data mining and machine learning arise as a key process providing insight into possible relationships among molecules and conditions such as gene–gene, protein–protein, drug–drug, drug–disease or gene–disease, etc.

From the perspective of DM, although there are several types of diabetes, the overall results suggest that the articles reviewed refer to T1D and T2D, with T2D representing the majority of the articles. A few articles refer to prediabetes and only one pertains to the metabolic syndrome, which is a term for metabolism-related pathophysiology. The types of data used in each case of the present collection were either clinical, genetic, electrochemical, chemical or medical. Only a few articles used clinical data in combination with genetic data. In addition, it is worth mentioning that the vast majority of the articles reviewed handled only clinical datasets. When it comes to prediction, the main biomarkers used involve anthropometric parameters, demographic characteristics, known risk factors, medical and drug history data, laboratory measurements, and epidemiological data. The most common biomarker seems to be blood glucose levels (HbA1c), as expected, since its detection is the basic step toward diagnosis and classification of a candidate diabetic patient.

With regard to DM treatment, the articles associated with drugs and therapy cover several fields of interest that include a) medication prescriptions, b) dosage planning with emphasis on insulin administration, c) potential side-effects of medications non-related to the disease (e.g. statins), and d) prediction of personalized glycemic response following anti-diabetic medication. Only Shoombautong et al., in [119], deal with the discovery of novel anti-diabetic agents. Therefore, to our knowledge there is much work ahead to be done on drug and therapeutic protocol design as far as evaluation and data mining on already known blood glucose lowering factors, such as metformin.

Concerning the genetic background in DM and environmental factors affecting the onset and progression of the disease, it is worth noting that the present account presents an evident gap in research on diabetes with respect to data mining and machine learning. The articles reviewed employ the HLA gene complex, in relation to T1D, whereas the rest of them attempt to predict associations of pleiotropic genes with DM. Interestingly, Lopes et al. tried to associate two known genes with DM, following wet lab validation of the extracted information [132]. Finally, although SNPs are one of the most common genetic markers in various research fields, in the present study only two articles utilized SNPs to predict DM. As more genes involved in the pathogenesis of diabetes are gradually identified, it will become easier to gain deeper understanding of the mechanisms responsible for the disease development and progression. That can lead to new insights into the genetic epidemiology of diabetes and nature of gene–gene and gene–environment interactions.

Finally, Diabetic complications covered in the present study include nephropathy, Alzheimer's disease, diabetic foot, liver cancer, hypoglycemic events, heart disease, depression, and retinopathy. The majority of the articles deal with retinopathy. One plausible explanation, apart from the impact of the disease, could be the availability of data resources from routine clinical practice that allow information extraction.

## 7 Conclusions

In this study, a systematic effort was made to identify and review machine learning and data mining approaches applied on DM research. DM is rapidly emerging as one of the greatest global health challenges of the 21st century. To date, there is a significant work carried out in almost all aspects of DM research and especially biomarker identification and prediction-diagnosis. The advent of biotechnology, with the vast amount of data produced, along with the increasing amount of EHRs is expected to give rise to further in-depth exploration toward diagnosis, etiopathophysiology and treatment of DM through employment of machine learning and data mining techniques in enriched datasets that include clinical and biological information.

## Figures and Tables

**Table 1:** Comparison of different ML algorithms.

Publication	Type of DM	Type of data	No. of subjects	Compared algorithms	Validation method	Best accuracy
Cai et al. [27]	T2D	Gut microbiota	Dataset A: 344 Dataset B: 145	Logistic regression (LR), linear discriminant analysis (LDA), naïve Bayes (NB) and support vector machine (SVM)	10-fold cross-validation	SVM on several different experiments
Malik et al. [46]	Both types (hyperglycemia)	Electrochemical measurements of saliva	175	Logistic regression (LR), support vector machine (SVM) and artificial neural network (ANN)	3-fold cross-validation	SVM ACC = 84.09
Farran et al. [60]	T2D	Demographic, anthropometric, vital signs, diagnostic and clinical laboratory measurements	10,632	Logistic regression (LR), k-nearest neighbors (k-NN), multifactor dimensionality reduction (MDR) support vector machines (SVM)	5-fold cross-validation	SVM ACC = 81.3
Mani et al. [61]	T2D	Demographic, clinical lab values	2280 distributed in three datasets	Gaussian Naïve Bayes (NB), Logistic Regression (LR), K-nearest neighbor (k-NN, CART, Random Forests (RF), Support Vector Machine (SVM)	5-fold cross-validation	RF AUC = 0.803/0.807/0.877
Tapak et al. [141]	Nonspecific	Demographic, anthropometric, diagnostic and clinical laboratory measurements	6500	Artificial neural networks (ANN), support vector machines (SVM), fuzzy c-mean, Random Forests (RF)	10-fold cross-validation	SVM ACC = 0.986 AUC = 0.979
